# Supplementary material for: Exploring adult glioma through MRI: A review of publicly available datasets to guide efficient image analysis
Source: Neurooncol Adv. 2025 Jan 28;7(1):vdae197. doi: 10.1093/noajnl/vdae197 (PMC11773385; doi:10.1093/noajnl/vdae197)
Supplement: vdae197_suppl_Supplementary_Figures_S1-S2 [file vdae197_suppl_supplementary_figures_s1-s2.docx]

Supplementary material

Figure S1: logarithmic scale for visualization purposes. BRSA has the smallest size with less than 1 GB (903 MB), while the largest dataset of the study is UGBM with a size of 358 GB.

10000

Size (GB) Patient number

1000

100

10

1

0,1

Dataset

BRSA

BTC1

IRAD

DLGG

TRTR

LGGD

BTUP

BTC2

BITE

RIDN

REMB

BRAG

RMND

RGBM

GLRT

QBDM

LUMI

QGTR

CGBM

TLGG

EGD

ADMB

TGBM

AFMB

IGAP

UPDG

UGBM


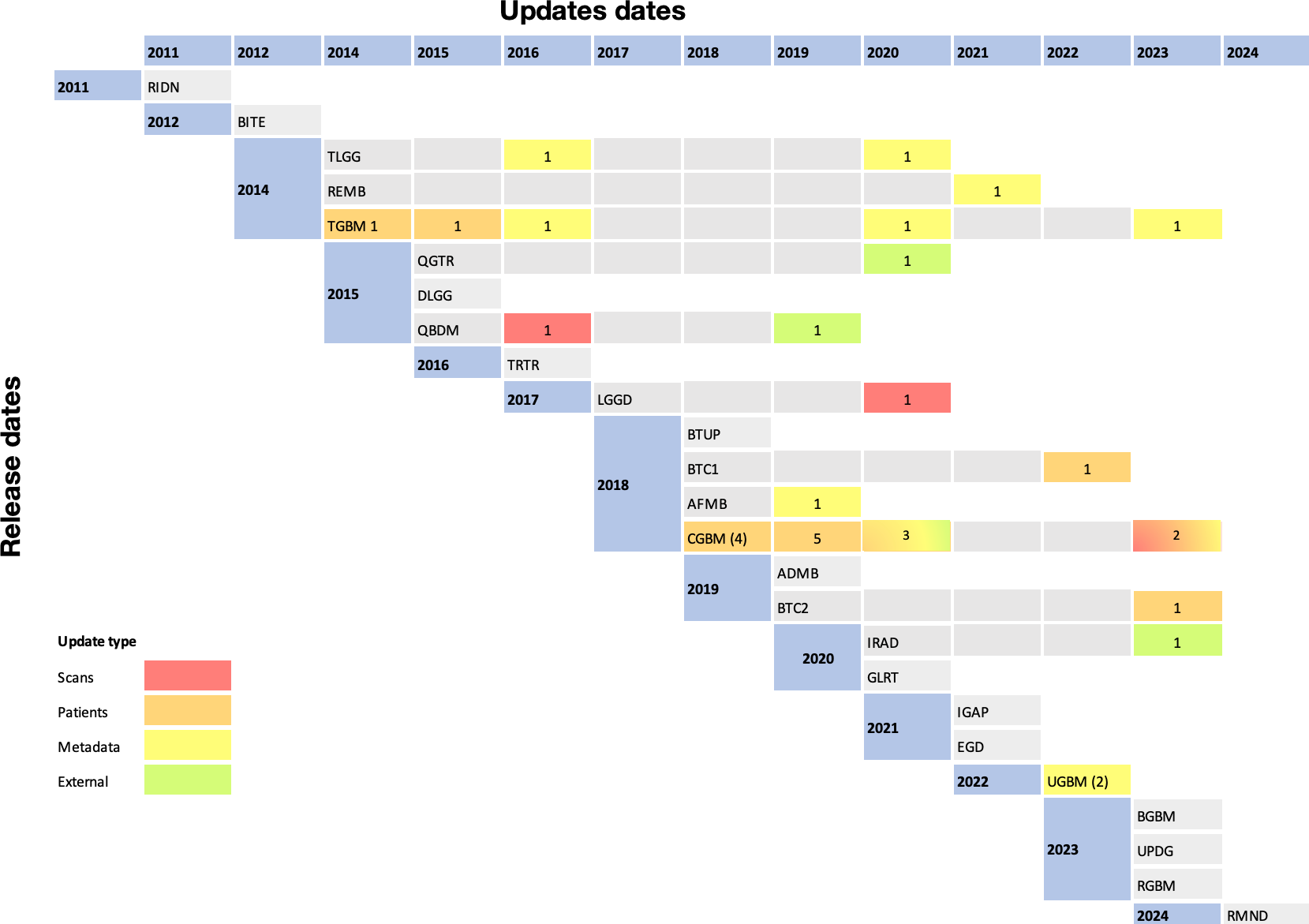


Figure S2: Classiﬁcation of datasets based on their release and update dates. Note the absence of new dataset releases in 2013. The table provides the frequency of updates per dataset annually, employing a color-coded system for clarity. A select number of datasets underwent multiple types of updates within a single year; for instance, the CGBM dataset received updates related to patients, metadata, and external sources in 2020, and updates concerning scans and patients in 2023.
